# Association between hemoglobin A1c levels and coronary flow reserve in angina with nonobstructive coronary arteries: a retrospective observational study

**DOI:** 10.1186/s12872-026-06449-6

**Published:** 2026-08-10

**Authors:** Keisuke Shirasaki, Takanori Ishida, Kentaro Niiya, Keisuke Kawatsu, Ryosuke Itakura, Kosuke Minai, Takayuki Ogawa, Michifumi Tokuda

**Affiliations:** 1 Department of Internal Medicine, Division of Cardiology, The Jikei University School of Medicine, 3-19-18 Nishi-Shimbashi, Minato-ku, Tokyo Japan; 2 Department of Internal Medicine, Division of Cardiology, The Jikei University School of Medicine, Katsushika Medical Center, Tokyo, Japan

**Keywords:** Angina with nonobstructive coronary arteries, Coronary microvascular dysfunction, Coronary flow reserve, Index of microvascular resistance, Hemoglobin A1c

## Abstract

**Background:**

Hemoglobin A1c (HbA1c) measures chronic glycemic levels, and its elevation is associated with vascular endothelial dysfunction and microcirculatory impairment, contributing to increased cardiovascular risk. In the assessment of angina with nonobstructive coronary arteries (ANOCA), coronary flow reserve (CFR) reflects vasodilatory capacity of the coronary microvasculature and is associated with adverse cardiovascular events, whereas IMR quantifies microvascular resistance during hyperemia. However, the association between HbA1c levels and invasive microvascular indices in ANOCA remains unclear.

**Methods:**

This study included 104 patients with ANOCA who underwent invasive coronary physiological assessment. CFR and IMR were measured using the thermodilution method. Associations between admission clinical parameters, including demographic characteristics and laboratory variables (particularly HbA1c), and CFR or IMR were examined using univariate and multivariate analyses.

**Results:**

The study population had a mean age of 65.8 ± 13.0 years, 50.0% were male, mean HbA1c was 6.0 ± 0.7%, mean CFR was 4.3 ± 2.4, and mean IMR was 14.2 ± 8.8. Multivariate analysis demonstrated a significant negative association between HbA1c and CFR (β = −0.250, 95% CI: −1.619 to − 0.155, *P* = 0.018). In contrast, no significant association was observed between HbA1c and IMR.

**Conclusions:**

In patients with ANOCA, higher HbA1c levels were independently associated with reduced CFR but not with IMR. These findings suggest a potential association between chronic glycemic status and coronary vasodilatory function in ANOCA.

**Supplementary Information:**

The online version contains supplementary material available at https://doi.org/10.1186/s12872-026-06449-6.

## Background

 Hemoglobin A1c (HbA1c) is a widely used metabolic marker that reflects average blood glucose levels over the past 1–2 months and serves as a cornerstone of diabetes management. Numerous epidemiological studies have demonstrated that elevated HbA1c is associated with an increased cardiovascular risk even among individuals without overt diabetes [1, 2].

Chronic hyperglycemia leads to vascular endothelial dysfunction through various mechanisms, including increased oxidative stress and activation of inflammatory pathways, resulting in reduced bioavailability of endothelial nitric oxide (NO). These processes disrupt blood flow regulation at the microvascular level [3–5]. Importantly, accumulating evidence suggests that microvascular dysfunction may develop even at the mild hyperglycemia stage, when glucose levels remain within the normal upper limit range. This raises the possibility that mild glucose abnormalities may adversely affect the coronary microvasculature [6–8].

Coronary flow reserve (CFR) and the index of microcirculatory resistance (IMR) are established indices for evaluating coronary microvascular function. CFR is defined as the ratio of coronary blood flow during maximal perfusion to that at rest, and a reduced CFR generally reflects impaired vasodilatory capacity of the coronary microvasculature. In contrast, IMR quantifies microvascular resistance during maximal hyperemia and is regarded as a more specific measure of microvascular resistance [9, 10]. These indices enable the diagnosis of coronary microvascular dysfunction (CMD) that cannot be identified via coronary angiography. Notably, reduced CFR has been reported to be independently associated with adverse cardiovascular events [11–13]. Angina with nonobstructive coronary arteries (ANOCA) has received increasing attention as a distinct clinical entity characterized by the presence of anginal symptoms in the absence of significant coronary artery stenosis. In patients with ANOCA, endothelial dysfunction and CMD are frequently observed and are recognized as key determinants of symptom burden and prognosis [14, 15].

Large international cohort studies, such as the COVADIS study, have increasingly recognized CMD as a key mechanism in patients with ANOCA [16]. Recent invasive physiological studies, including the ILIAS registry, have further advanced the understanding of coronary microvascular endotypes [17, 18].

Given these observations, a close association between glycemic status, as reflected by HbA1c, and coronary microvascular function is biologically plausible. However, studies simultaneously evaluating the association of HbA1c with both CFR and IMR in ANOCA patients remain limited. Moreover, only a few studies have rigorously examined whether HbA1c is independently associated with CMD after adjustment for potential confounders, including systemic inflammation, renal function, and lipid parameters. Therefore, the potential impact of mildly elevated HbA1c on coronary microvascular function in this population remains insufficiently defined.

Accordingly, this retrospective observational study aimed to investigate the association between HbA1c levels and coronary microvascular function in patients with ANOCA. Using univariate and multivariate analyses, we tested the hypothesis that elevated HbA1c is independently associated with reduced CFR in ANOCA patients. In addition, we evaluated the relationship between HbA1c and IMR to further characterize their associations with coronary microvascular function.

## Methods

### Patient population

This retrospective observational study enrolled patients with ANOCA who were hospitalized at Jikei University Katsushika Medical Center between November 2023 and October 2025 and underwent coronary angiography. ANOCA was defined as the presence of anginal symptoms without obstructive coronary artery disease. Obstructive coronary artery disease was defined as ≥ 50% diameter stenosis on coronary angiography or functionally significant stenosis with a fractional flow reserve (FFR) ≤ 0.80 [15, 19–22]. During the study period, 131 patients were suspected of having ANOCA and underwent coronary angiography. Patients were excluded if they met any of the following criteria: (1) maintenance hemodialysis (*n* = 2), (2) atrial fibrillation (*n* = 6), (3) heart failure (HF) (*n* = 4), or (4) noncardiac chest pain (*n* = 15). Based on these selection criteria, 104 patients were enrolled in the final analysis. HF was defined as a pulmonary capillary wedge pressure of ≥ 18 mmHg measured using a Swan–Ganz catheter.

The study protocol was approved by the Ethics Committee of Jikei University School of Medicine (Approval No. 24–355[7121]) and was conducted in accordance with the institutional ethical standards and the principles of the Declaration of Helsinki. Owing to the retrospective nature of the study, informed consent was not obtained. Instead, details regarding the study design and contact information were made publicly available at the institution, and patients were provided the opportunity to decline participation through an opt-out process.

### Data collection

All data were obtained from medical records. Clinical and laboratory variables at admission included age, sex, body mass index (BMI), mean blood pressure, serum creatinine (Cr), C-reactive protein (CRP), low-density lipoprotein cholesterol (LDL-C), and HbA1c. Acetylcholine provocation testing was performed according to operator judgment and institutional practice. Accordingly, not all patients underwent acetylcholine provocation testing, as the decision to perform the test was left to the discretion of the attending physician. In patients who underwent acetylcholine provocation testing, the test was routinely performed before the assessment of CFR and IMR according to our institutional protocol. CFR and IMR were then assessed using a pressure–temperature sensor guidewire and the bolus thermodilution method. CFR and IMR were measured in the left anterior descending artery (LAD) in all patients. The mean transit time was determined by repeated injection of room-temperature saline. Maximal coronary hyperemia was induced by intracoronary administration of nicorandil (2 mg). In the previous validation study by Jang et al., nicorandil was recognized as the most effective alternative to adenosine for hyperemia and was used [23]. CFR was calculated as the ratio of mean transit time at rest to mean transit time during maximum hyperemia, while the IMR was calculated as the product of distal coronary artery pressure and mean transit time during maximal hyperemia. CMD was defined as CFR < 2.0 and IMR ≥ 25. Epicardial coronary spasm (CSA) was diagnosed when acetylcholine provocation induced ≥ 90% transient epicardial coronary constriction accompanied by anginal symptoms and ischemic electrocardiographic changes. Microvascular spasm (MVS) was diagnosed when acetylcholine provocation induced anginal symptoms and ischemic electrocardiographic changes without significant epicardial coronary spasm, according to the Japanese Circulation Society recommendations [9, 24].

### Statistical analyses

Continuous variables are presented as mean ± standard deviation (SD), and categorical variables as counts and percentages. No missing data were observed for the variables included in the analyses. The distribution of HbA1c was assessed by visual inspection of Q–Q plots. The linearity of the association between HbA1c and CFR was assessed by including a quadratic term (HbA1c²) in the regression model. CRP showed a mildly right-skewed distribution; therefore, additional analyses were performed using log-transformed values. As a supplementary analysis, patients were stratified into three groups according to HbA1c levels: normal (< 5.7%), prediabetes (5.7–6.4%), and diabetes (≥ 6.5%). Differences in CFR among the groups were assessed using the Kruskal–Wallis test, followed by Bonferroni-adjusted post hoc pairwise comparisons.

Univariate analyses were performed to examine associations between clinical and laboratory variables and CFR and IMR. Covariates included in the multivariable regression model (age, sex, body mass index, mean blood pressure, serum creatinine, C-reactive protein, LDL cholesterol, and HbA1c) were selected based on their established clinical relevance to coronary microvascular function and biological plausibility. Given the relatively small sample size, the number of covariates was intentionally limited to reduce the risk of overfitting.

Additional exploratory sensitivity analyses were performed to assess the robustness of the primary findings. These included sensitivity analyses excluding the three insulin-treated patients and patients with overt diabetes, as well as sex interaction, sex-stratified, FFR-adjusted, and medication-adjusted analyses. To assess whether the association between HbA1c and CFR was influenced by overt diabetes mellitus, we conducted a sensitivity analysis excluding individuals with HbA1c ≥ 6.5% or a known history of diabetes. Multivariable linear regression analysis was repeated in this restricted cohort using the same covariates as in the primary model.

To evaluate whether the association between HbA1c and CFR differed by sex, an interaction term (HbA1c × sex) was included in the multivariable regression model. HbA1c was mean-centered before creating the interaction term to reduce multicollinearity. In addition, sex-stratified analyses were performed using separate multivariable regression models in men and women. To assess the potential influence of residual epicardial physiological disease, an additional multivariable regression analysis including FFR was performed in patients with available FFR data.

To assess the potential confounding effects of medications, patients were stratified into tertiles according to HbA1c levels. Differences in medication use across tertiles were evaluated using the chi-square test. Associations between medication use and CFR were examined using linear regression analysis. Furthermore, partial correlation analysis was performed to assess the relationship between HbA1c and CFR after adjustment for medication use (including statins, calcium channel blockers, β-blockers, and renin–angiotensin system inhibitors).

Statistical analyses were performed using IBM SPSS version 31 (SPSS Inc., Chicago, IL, USA). A two-sided *P* value < 0.05 was considered to indicate statistical significance.

Clinical trial number: Not applicable.

## Results

### Characteristics of the study population

The clinical characteristics of the study population (*n* = 104) are summarized in Table 1. The mean age was 65.8 ± 13.0 years, with 50.0% being male. The mean HbA1c at admission was 6.0% ± 0.7 (range, 5.0–8.0%), mean CFR was 4.3 ± 2.4, and mean IMR was 14.2 ± 8.8. The distribution of HbA1c was approximately normal on visual inspection of Q–Q plots, with only minor deviations at the extremes. The quadratic term (HbA1c²) was not significant (*P* = 0.639).

**Table 1:** Characteristics of the study population

Variable (*n* = 104)	Value, Mean ± (SD), Number (%)
Men (%)	52 (50.0)
Age	65.8 (13.0)
Height (cm)	161.1 (8.6)
Weight (kg)	65.1 (15.5)
BMI (kg/m^2^)	24.9 (4.7)
Mean blood pressure (mmHg)	106.7 (15.8)
WBC count (×10^3^/µL)	6.2 (1.9)
Hb (g/dL)	13.5 (1.6)
PLT count (×10^3^/µL)	219.4 (51.2)
Cr (mg/dL)	0.82 (0.23)
UA (mg/dL)	5.4 (1.6)
BNP (pg/mL)	86.8 (205.8)
CRP (mg/dL)	0.2 (0.3)
LDL-C (mg/dL)	106.5 (35.7)
HbA1c (%)	6.0 (0.7)
CFR	4.3 (2.4)
IMR	14.2 (8.8)
Underlying disease	
Hypertension	63 (73.6)
Diabetes mellitus	17 (16.3)
Dyslipidemia	51 (49.0)
Smoking history	
Current smoker	14 (13.5)
Past smoker	26 (25.0)
Never smoker	64 (61.5)
Medication	
Calcium channel blockers	35 (33.7)
Beta blockers	25 (24.0)
ACE inhibitors	3 (2.9)
ARBs	29 (27.9)
ARNIs	10 (9.6)
Oral antidiabetic agents	9 (8.7)
Insulin	3 (2.9)
Statins	45 (43.3)
Coronary vasodilators	29 (27.9)
Diuretics	19 (18.3)
Antiplatelet drugs	22 (21.2)
Anticoagulant agents	9 (8.7)

*ACE* angiotensin-converting enzyme, *ARB* angiotensin II type I receptor blocker, *ARNI* angiotensin receptor neprilysin inhibitor, *BMI* body mass index, *BNP* B-type natriuretic peptide, *CFR* coronary flow reserve, *Cr* creatinine, *CRP* C-reactive protein, *Hb* hemoglobin, *HbA1c* hemoglobin A1c, *IMR* index of microcirculatory resistance, *LDL-C* low-density lipoprotein cholesterol, *PLT* platelet, *UA* uric acid, *WBC* white blood cell

The ANOCA endotypes are summarized in Supplementary Table S1. Acetylcholine provocation testing was performed in 73 patients (70.2%). Coronary spasm alone was diagnosed in 58 patients, microvascular spasm alone in 1 patient, combined coronary spasm and CMD in 2 patients, and CMD alone in 4 patients. Overall, 39 patients could not be assigned a definitive functional diagnosis. Reduced CFR (CFR < 2.0) and elevated IMR (IMR ≥ 25) were observed in 12 (11.5%) and 15 (14.4%) patients, respectively.

As a supplementary analysis, CFR was compared across HbA1c categories (normal, prediabetes, and diabetes). No significant difference in CFR was observed among the three groups (Kruskal–Wallis test, *P* = 0.128; Supplementary Figure S1). Bonferroni-adjusted post hoc pairwise comparisons showed no significant differences between any groups.

### Regression analysis

**Fig. 1:**
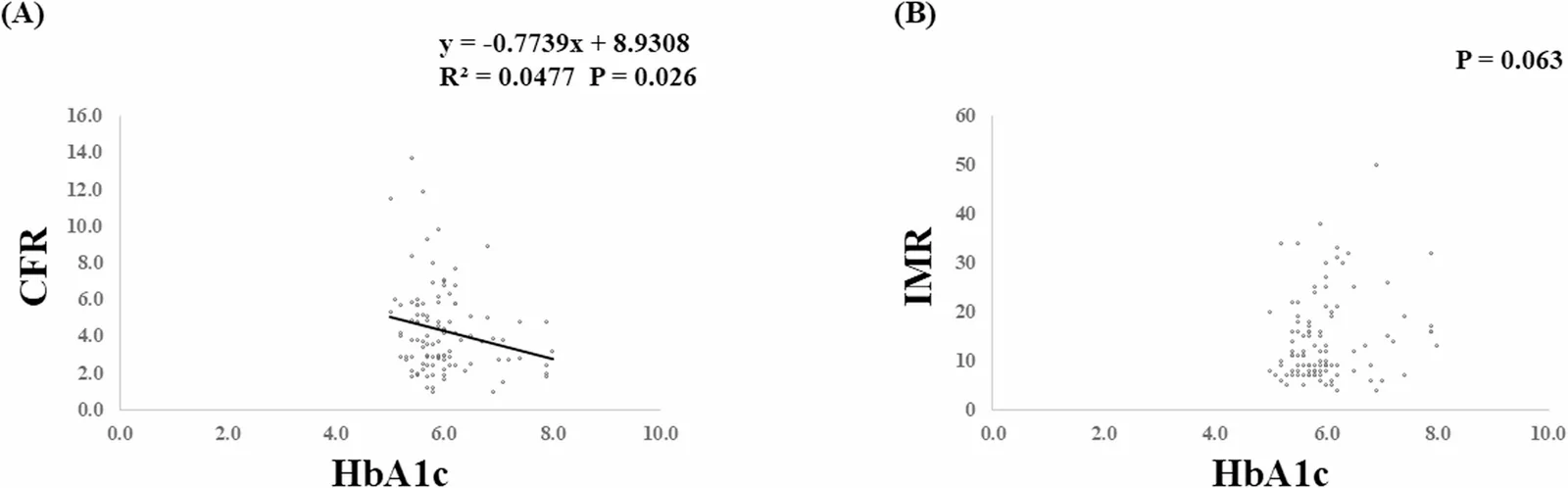
Associations between HbA1c and (**A**) CFR and (**B**) IMR. Scatter plots from univariate regression analyses illustrating the relationships between admission HbA1c and CFR and IMR. Abbreviations: CFR, coronary flow reserve; HbA1c, hemoglobin A1c; IMR, index of microcirculatory resistance

The results of the univariate regression analyses are presented in Tables 2 and 3, and the corresponding scatter plots are shown in Fig. 1, Supplementary Figure S2 and S3. CFR showed a significant negative association with HbA1c at admission (β = −0.218, *P* = 0.026). No other clinical or laboratory variables were significantly associated with CFR in univariate analyses. Conversely, IMR showed significant positive associations with age (β = 0.210, *P* = 0.032) and mean blood pressure at admission (β = 0.204, *P* = 0.037).

**Table 2:** Univariate and multiple regression analysis for CFR and IMR. Univariate regression analysis

	Regression coefficient	Standard error	Standard regression coefficient	Test statistic	*P* value	95% confidence interval
Age	−0.032	0.018	−0.177	−1.814	0.073	−0.068 to 0.003
Sex	0.210	0.465	0.045	0.450	0.653	−0.714 to 1.133
BMI	0.052	0.049	0.103	1.047	0.298	−0.046 to 0.150
Mean blood pressure	0.010	0.015	0.069	0.694	0.489	−0.019 to 0.040
WBC count	0.014	0.123	0.012	0.118	0.907	−0.229 to 0.258
Hb	0.130	0.148	0.087	0.882	0.380	−0.163 to 0.423
PLT count	−0.003	0.005	−0.062	−0.627	0.532	−0.012 to 0.006
Cr	0.290	1.033	0.028	0.281	0.780	−1.759 to 2.338
UA	0.183	0.147	0.123	1.247	0.215	−0.108 to 0.475
BNP	0.000	0.001	−0.022	−0.227	0.821	−0.003 to 0.002
CRP	−0.718	0.757	−0.093	−0.948	0.345	−2.219 to 0.784
LDL-C	−0.003	0.007	−0.049	−0.491	0.625	−0.016 to 0.010
HbA1c	−0.774	0.343	−0.218	−2.259	0.026	−1.453 to −0.094
Objective variable: CFR						
Explanatory variables: Age, Sex, BMI, Mean blood pressure, WBC count, Hb, PLT count, Cr, UA, BNP, CRP, LDL-C, and HbA1c						
Age	0.142	0.065	0.210	2.174	0.032	0.012–0.272
Sex	0.250	1.727	0.014	0.145	0.885	−3.175 to 3.675
BMI	0.177	0.183	0.095	0.966	0.336	−0.187 to 0.541
Mean blood pressure	0.114	0.054	0.204	2.109	0.037	0.007 to 0.221
WBC count	0.429	0.452	0.094	0.949	0.345	−0.468 to 1.326
Hb	0.425	0.547	0.077	0.776	0.439	−0.661 to 1.511
PLT count	−0.022	0.017	−0.127	−1.288	0.201	−0.055 to 0.012
Cr	3.922	3.809	0.101	1.030	0.306	−3.633 to 11.477
UA	0.775	0.543	0.140	1.426	0.157	−0.303 to 1.853
BNP	−0.008	0.004	−0.181	−1.859	0.066	−0.016 to 0.001
CRP	−5.061	2.772	−0.178	−1.826	0.071	−10.559 to 0.437
LDL-C	0.004	0.024	0.018	0.184	0.854	−0.044 to 0.053
HbA1c	2.400	1.279	0.183	1.877	0.063	−0.136 to 4.937
Objective variable: IMR						
Explanatory variables: Age, Sex, BMI, Mean blood pressure, WBC count, Hb, PLT count, Cr, UA, BNP, CRP, LDL-C, and HbA1c						

*BMI* body mass index, *BNP * B-type natriuretic peptide, *CFR* coronary flow reserve, *Cr* creatinine, *CRP* C-reactive protein, *Hb* hemoglobin, *HbA1c* hemoglobin A1c, *IMR* index of microcirculatory resistance, *LDL-C* low-density lipoprotein cholesterol, *PLT* platelet, *UA* uric acid, *WBC* white blood cell

**Table 3:** Univariate and multiple regression analysis for CFR and IMR. Multiple regression analysis

R^2^ = 0.132	Regression coefficient	Standard error	Standard regression coefficient	Test statistic	*P* value	95% confidence interval	VIF
Age	−0.029	0.019	−0.160	−1.501	0.137	−0.068 to 0.009	1.239
Sex	0.582	0.580	0.124	1.004	0.318	−0.569 to 1.733	1.663
BMI	0.091	0.055	0.180	1.639	0.105	−0.019 to 0.200	1.327
Mean blood pressure	0.005	0.016	0.034	0.326	0.745	−0.026 to 0.036	1.180
Cr	0.961	1.287	0.092	0.747	0.457	−1.593 to 3.516	1.665
CRP	−0.873	0.804	−0.114	−1.086	0.280	−2.470 to 0.723	1.202
LDL-C	−0.013	0.007	−0.202	−1.817	0.072	−0.028 to 0.001	1.351
HbA1c	−0.887	0.369	−0.250	−2.404	0.018	−1.619 to −0.155	1.185
Objective variable: CFR							
Explanatory variables: Age, Sex, BMI, Mean blood pressure, CRP, LDL-C, and HbA1c							
Age	0.130	0.070	0.193	1.852	0.067	−0.009 to 0.270	1.239
Sex	1.585	2.105	0.091	0.753	0.453	−2.594 to 5.764	1.663
BMI	0.233	0.201	0.125	1.160	0.249	−0.166 to 0.631	1.327
Mean blood pressure	0.083	0.057	0.148	1.460	0.148	−0.030 to 0.195	1.180
Cr	6.399	4.670	0.165	1.370	0.174	−2.872 to 15.671	1.665
CRP	−5.115	2.920	−0.180	−1.752	0.083	−10.911 to 0.682	1.202
LDL-C	0.006	0.027	0.026	0.242	0.809	−0.047 to 0.060	1.351
HbA1c	2.448	1.339	0.186	1.829	0.071	−0.209 to 5.106	1.185
Objective variable: IMR							
Explanatory variables: Age, Sex, BMI, Mean blood pressure, Cr, CRP, LDL-C, and HbA1c							

*BMI* body mass index, *CFR* coronary flow reserve, *Cr* creatinine, *CRP* C-reactive protein, *HbA1c* hemoglobin A1c, *IMR* index of microcirculatory resistance, *LDL-C* low-density lipoprotein cholesterol, *VIF* variance inflation factor

The results of multivariate regression analyses are summarized in Table 2 and 3. After adjustment for potential confounders, HbA1c at admission remained independently and negatively associated with CFR (β = −0.250, 95% CI: −1.619 to − 0.155, *P* = 0.018). No other variables demonstrated an independent association with CFR. In the multivariate model for IMR, none of the examined factors showed a statistically significant association.

CRP showed a mildly right-skewed distribution; therefore, additional analyses were performed using log-transformed values, which yielded consistent results. Accordingly, the primary analyses are presented using the original values for ease of interpretation.

Additional exploratory sensitivity analyses were performed to assess the robustness of the primary findings. The results of a multivariate analysis excluding three insulin users are shown in Supplementary Table S2. The results remained unchanged, with HbA1c at admission showing independently and negatively associated with CFR (β = −0.223, 95% CI: −1.632 to − 0.056, *P* = 0.036). Other variables did not show an independent association with CFR. The results were also unchanged in the IMR multivariate model, with none of the factors examined showing a statistically significant association.

A total of 83 patients remained after excluding those with overt diabetes mellitus. In this restricted cohort, HbA1c was not significantly associated with CFR in the multivariable analysis (β = −0.165, 95% CI: −3.193 to 0.620, *P* = 0.183) (Supplementary Table S3). FFR data were available for 100 patients (mean FFR 0.88 ± 0.04). In an additional multivariable regression analysis including FFR, HbA1c remained independently associated with CFR (β = −0.273, 95% CI: −1.718 to − 0.228, *P* = 0.011), whereas FFR was not significantly associated with CFR (β = 0.112, 95% CI: −5.859 to 19.36, *P* = 0.290) (Supplementary Table S4).

As an additional exploratory analysis, multivariable logistic regression using CFR < 2.5 as the outcome was performed. HbA1c was not independently associated with reduced CFR (OR 1.85, 95% CI: 0.82–4.16, *P* = 0.136) (Supplementary Table S5).

### Sex-stratified and interaction analysis

As an additional exploratory analysis, the association between HbA1c and CFR was evaluated according to sex. In the multivariable model including the interaction term (HbA1c × sex), HbA1c remained significantly associated with CFR (β = −0.251, 95% CI: −1.735 to − 0.044, *P* = 0.039), whereas no significant interaction was observed (P for interaction = 0.988) (Supplementary Table S6). In sex-stratified analyses, HbA1c was not significantly associated with CFR in either men (β = −0.199, 95% CI: −1.294 to 0.203, *P* = 0.149) or women (β = −0.122, 95% CI: −2.256 to 1.000, *P* = 0.442) (Supplementary Table S7 and S8).

### Assessment of potential confounding by medications

As an additional exploratory analysis, the potential confounding effects of medications were evaluated. Statin use differed significantly across HbA1c tertiles (χ² = 15.09, *P* < 0.001), whereas the use of calcium channel blockers (χ² = 1.01, *P* = 0.604), β-blockers (χ² = 2.81, *P* = 0.246), and RAS inhibitors (χ² = 2.78, *P* = 0.249) did not differ significantly across tertiles. In regression analyses, none of the medications were significantly associated with CFR (statins: β = −0.086, *P* = 0.424; calcium channel blockers: β = 0.113, *P* = 0.273; β-blockers: β = −0.052, *P* = 0.642; RAS inhibitors: β = 0.101, *P* = 0.344). Furthermore, the inverse association between HbA1c and CFR remained significant after adjustment for medication use (partial *r* = − 0.220, *P* = 0.028) (Supplementary Table S9).

## Discussion

### Summary of the results

Using comprehensive clinical variables and statistical approaches, this study demonstrated that HbA1c levels were significantly associated with CFR in patients with ANOCA undergoing invasive physiological testing. Consistent with our hypothesis, higher HbA1c levels showed a significant association with reduced CFR, suggesting that chronic glycemic status may be associated with coronary microvascular function. However, no significant association was observed between HbA1c and IMR.

### Pathophysiological basis of the relationship between HbA1c and CFR

HbA1c is a surrogate marker of chronic glycemic exposure rather than a direct causal factor. Therefore, the observed association likely reflects the cumulative effects of chronic hyperglycemia on coronary microvascular function rather than an effect of HbA1c itself. In this study, higher HbA1c levels at admission were associated with lower CFR, consistent with prior physiological evidence linking poor glycemic control to coronary microvascular dysfunction [25–27]. Chronic hyperglycemia induces a cascade of endothelial abnormalities that adversely affect microvascular function. Enhanced oxidative stress and activation of inflammatory pathways arise from metabolic disturbances such as increased polyol pathway flux, accumulation of advanced glycation end products, and activation of protein kinase C. These processes inhibit endothelial nitric oxide synthase activity, reduce NO bioavailability, and impair endothelium-dependent vasodilation in the coronary microvasculature.

Furthermore, chronic hyperglycemia enhances the activity of reactive oxygen species (ROS)–producing enzymes, including nicotinamide adenine dinucleotide phosphate oxidase, xanthine oxidase, and cyclooxygenase, thus exacerbating oxidative stress within vascular endothelial cells. Consequently, NO-dependent vasodilation responses are further attenuated [3, 28, 29]. These changes are associated with reduced CFR.

Furthermore, HbA1c does not capture postprandial hyperglycemia, glycemic variability, or insulin resistance, all of which have been implicated in endothelial dysfunction and coronary microvascular dysfunction. These metabolic abnormalities may also contribute to the observed association and should be investigated in future studies.

Although these processes may be modified by conventional cardiovascular risk factors such as blood pressure, renal function, and dyslipidemia, this study employed multivariate analysis to account for these confounding factors. This analytical approach supports an independent association between HbA1c and coronary microvascular function.

Notably, the observed association was present despite mean HbA1c levels of approximately 6.0%, which is below conventional diagnostic thresholds for diabetes. However, because the association was attenuated after excluding patients with overt diabetes, these findings should be interpreted with caution. Further studies are needed to clarify whether HbA1c is independently associated with coronary microvascular dysfunction in individuals without overt diabetes.

### Physiological interpretation of CFR and IMR

In this study, elevated HbA1c levels were independently associated with decreased CFR, while no association was observed with IMR. Traditionally, CFR has been categorized as a functional abnormality and IMR as a structural abnormality, but this may oversimplify the physiology underlying these indices. CFR is influenced by factors such as resting hemodynamics, heart rate, and aortic pressure and is therefore not purely “functional”. Similarly, IMR, although specific to the microvascular system, is not solely structural.

Recently, de Vos et al. and Nijkamp et al. proposed and validated an index known as microvascular resistance reserve (MRR) that corrects CFR for epicardial artery stenosis and hemodynamic fluctuations (blood pressure). Furthermore, it can more specifically assess the vasodilatory reserve of the microvasculature [17, 18]. Recent studies have demonstrated reduced MRR in patients with diabetes, suggesting impaired coronary microvascular vasodilatory reserve in this population [30]. Furthermore, accumulating evidence indicates that MRR is a physiologically robust and prognostically relevant index for the assessment of coronary microvascular function [31]. Because MRR could not be calculated in the present retrospective study, future investigations incorporating MRR may provide additional insights into the relationship between HbA1c levels and coronary microvascular function in patients with ANOCA.

According to the latest findings, CMD can be classified into two endotypes: (1) functional CMD, where microvascular resistance (IMR and hyperemic microvascular resistance) at maximal hyperemia is within the normal range but the vasodilation reserve (MRR and CFR) is reduced; and (2) structural CMD, where microvascular resistance at maximal hyperemia is abnormally elevated and the vasodilatation reserve is reduced [18].

In this study, higher HbA1c levels were independently associated with decreased CFR, while no significant association was observed with IMR. This suggests that hyperglycemia may be associated with a decrease in vasodilation reserve (functional CMD) before causing structural remodeling of microvascular vessels (structural CMD). However, considering that CFR is influenced by resting hemodynamics, heart rate, aortic pressure, and residual epicardial coronary disease and therefore does not exclusively reflect coronary microvascular vasodilatory function. In contrast, MRR corrects for both epicardial disease and hemodynamic conditions, providing a more specific assessment of coronary microvascular function [32]. Accordingly, the observed association between HbA1c and CFR, but not IMR, should not be interpreted as direct evidence of impaired hyperemic microvascular vasodilatory capacity. Instead, it may reflect alterations in resting coronary blood flow, hyperemic coronary blood flow, or both. Future research should further investigate the effects of hyperglycemia on microvascular endotypes using more specific indicators such as MRR.

### Discrepancies with prior studies

Although this study corroborated the conventional view that elevated HbA1c is associated with decreased CFR, our findings differ from the findings of Teragawa et al. (2024) in Japanese patients with ANOCA [33]. In this previous study, lower HbA1c levels were associated with CMD in women, whereas no significant association was observed in the overall cohort or in men.

Several factors may explain this discrepancy. First, differences in coronary spasm provocation testing (SPT) may be relevant. In the study by Teragawa et al., SPT using acetylcholine and ergonovine was systematically performed prior to CFR/IMR measurement in all patients, while in the present study, acetylcholine testing was performed in 73 of the 104 patients (70.2%). The remaining patients did not undergo vasomotor testing. Consequently, patients with vasospastic angina may have been included without being identified, potentially introducing unmeasured confounding. Second, differences in hyperemia-inducing agents may have contributed to this discrepancy. The present study used nicorandil, whereas Teragawa et al. used ATP. Differences in pharmacological mechanisms may have contributed to variations in physiological measurements. Third, differences in glycemic profiles may be relevant. Although mean HbA1c levels were similar between studies (approximately 6.0%), the proportion of patients with diabetes was higher in the present study than in the study by Teragawa et al. Such differences in patient characteristics may have influenced the observed associations. Finally, the trend reported by Teragawa et al. may reflect a chance finding in a relatively small female subgroup, as acknowledged by the authors themselves.

In contrast, our findings are in line with recent evidence demonstrating an association between chronic hyperglycemia and CMD using alternative indices (e.g., angiographic microvascular resistance) [34].

The recent ESC Working Group statement emphasizes that coronary microvascular dysfunction may differ between women and men with respect to pathophysiology, clinical presentation, and underlying mechanisms [35]. Previous studies have suggested potential sex-related variability in CMD. However, no significant interaction between HbA1c and sex was observed in the present study, and no significant association was identified in sex-stratified analyses. Despite the limited statistical power, these findings suggest that the association between HbA1c and CFR is not strongly modified by sex.

The mean CFR in the present study (4.3) was higher than that reported in prior ANOCA studies (typically 2–3.5 using bolus thermodilution). This difference may be explained by variations in patient selection, measurement techniques, and hyperemic protocols as well as inherent variability in thermodilution-based measurements. In addition, intracoronary nicorandil was used to induce hyperemia in the present study. Although nicorandil has been validated as an effective alternative to adenosine for coronary physiological assessment [23], physiological measurements obtained using different hyperemic agents may not be directly comparable. Furthermore, papaverine has also been established as a clinically validated hyperemic agent for invasive physiological assessment [36]. Therefore, caution is warranted when directly comparing CFR values across studies.

### Significance of this study

In patients with ANOCA, CMD is an important determinant of symptoms, quality of life, and prognosis. This study suggests that HbA1c, a readily available and routinely measured biomarker, may be associated with coronary microvascular function in patients with ANOCA.

Previous studies have demonstrated associations between glycemic abnormalities and coronary microvascular dysfunction using both invasive and non-invasive assessments. The present study extends these findings by evaluating the associations of HbA1c with both CFR and IMR using invasive physiological assessment in patients with ANOCA. Notably, HbA1c was independently associated with CFR but not IMR, providing additional insight into the relationship between chronic hyperglycemia and coronary microvascular function.

These findings are consistent with the concept that hyperglycemia-related abnormalities in coronary microvascular function may initially manifest as impaired vasodilatory capacity. Notably, the mean HbA1c level in the present cohort was approximately 6.0%. However, because the association between HbA1c and CFR was attenuated after excluding patients with overt diabetes, further studies are required to determine whether similar associations are present in individuals without overt diabetes. Future interventional studies are warranted to clarify whether improved glycemic control is associated with changes in CFR.

### Limitations

This study has several limitations. First, in sensitivity analyses, the association between HbA1c and CFR remained significant after excluding insulin-treated patients; however, it was attenuated and no longer significant after excluding patients with overt diabetes, although the direction of the association remained consistent. These findings suggest that the observed association may be partly influenced by higher glycemic ranges; however, this result should be interpreted cautiously given the reduced statistical power in the restricted cohort. Second, the relatively small sample size and single-center retrospective design may limit the generalizability of the findings. Third, although medication use (including statins, calcium channel blockers, β-blockers, and renin–angiotensin system inhibitors) was adjusted for in multivariate and sensitivity analyses, residual confounding effects cannot be completely excluded. However, the consistent results after adjustment suggest that the observed association is unlikely to be substantially driven by concomitant medications. Fourth, the relatively low R² values observed in this study indicate that a substantial portion of CFR and IMR variability remains unexplained, suggesting that coronary microvascular function is influenced by multiple factors. Therefore, HbA1c should be interpreted as one of several contributing factors rather than a sole determinant of coronary microvascular function. Fifth, other metabolic parameters such as fasting blood glucose, postprandial glucose spikes, indices of insulin resistance (e.g., homeostatic model assessment of insulin resistance), and high-density lipoprotein cholesterol were not evaluated, precluding a more detailed examination of the underlying metabolic mechanisms. Furthermore, HbA1c reflects average glycemic exposure but does not capture postprandial glucose spikes or insulin resistance, both of which may be associated with oxidative stress and early CMD. Sixth, technical reproducibility metrics, such as the coefficient of variation or intraclass correlation coefficient, were not evaluated because repeated physiological measurements were not systematically performed in this retrospective study. Therefore, measurement variability could not be formally assessed. Seventh, hyperemia was induced using intracoronary nicorandil rather than adenosine or ATP. Therefore, the absolute values of CFR and IMR may not be fully comparable with those obtained using standard adenosine-based protocols. Finally, acetylcholine provocation testing was not performed in all patients because the decision to perform the test was left to the discretion of the attending physician. Therefore, potential selection bias related to incomplete vasomotor assessment cannot be excluded.

The present findings should therefore be considered hypothesis-generating and require confirmation in larger prospective multicenter studies.

## Conclusions

In patients with ANOCA, higher HbA1c levels were independently associated with reduced CFR but not with IMR. These findings suggest a potential association between chronic glycemic status and coronary vasodilatory function in ANOCA.

## Supplementary Information

Supplementary Material 1: Supplementary Fig. S1. Distribution of coronary flow reserve according to HbA1c categories. Box-and-whisker plots show the distribution of CFR according to HbA1c categories: normal HbA1c (<5.7%), prediabetes (5.7–6.4%), and diabetes (≥6.5%). Differences among the three groups were assessed using the Kruskal–Wallis test, followed by Bonferroni-adjusted pairwise comparisons. Abbreviations: CFR, coronary flow reserve; HbA1c, hemoglobin A1c.

Supplementary Material 2: Supplementary Fig. S2. Associations between admission variables and CFR. Scatter plots from univariate regression analyses illustrating the relationships between admission clinical parameters (age, sex, BMI, mean arterial blood pressure, WBC count, Hb, PLT count, Cr, UA, BNP, CRP, and LDL-C) and CFR (12 plots). Abbreviations: BMI, body mass index; BNP, B-type natriuretic peptide; CFR, coronary flow reserve; Cr, creatinine; CRP, C-reactive protein; Hb, hemoglobin; IMR, index of microcirculatory resistance; LDL-C, low-density lipoprotein cholesterol; PLT, platelet; UA, uric acid; WBC, white blood cell.

Supplementary Material 3: Supplementary Fig. S3. Associations between admission variables and IMR. Scatter plots from univariate regression analyses illustrating the relationships between admission clinical parameters (age, sex, BMI, mean arterial blood pressure, WBC count, Hb, PLT count, Cr, UA, BNP, CRP, and LDL-C) and IMR (12 plots). Abbreviations: BMI, body mass index; BNP, B-type natriuretic peptide; CFR, coronary flow reserve; Cr, creatinine; CRP, C-reactive protein; Hb, hemoglobin; IMR, index of microcirculatory resistance; LDL-C, low-density lipoprotein cholesterol; PLT, platelet; UA, uric acid; WBC, white blood cell.

Supplementary Material 4.

## Data Availability

The datasets generated and/or analyzed during the current study are available from the corresponding author on reasonable request.

## Abbreviations

ANOCA: angina with nonobstructive coronary arteries
BMI: body mass index
CFR: coronary flow reserve
CMD: coronary microvascular dysfunction
Cr: Creatinine
CRP: C-reactive protein
HbA1c: FFR: fractional flow reserve, hemoglobin A1c
HF: Heart failure
IMR: Index of microcirculatory resistance
LDL-C: Low-density lipoprotein cholesterol
MRR: Microvascular resistance reserve
NO: Nitric oxide
ROS: Reactive oxygen species
SPT: Spasm provocation testing
