# Supplementary material for: Association between hemoglobin A1c levels and coronary flow reserve in angina with nonobstructive coronary arteries: a retrospective observational study
Source: BMC Cardiovasc Disord. 2026 Aug 10;26:1038. doi: 10.1186/s12872-026-06449-6 (PMC13741783; doi:10.1186/s12872-026-06449-6)
Supplement: Supplementary file 1 [file 12872_2026_6449_MOESM4_ESM.zip › Revised Supplementary Table S4.docx]

**Supplementary Table S4. Multiple regression analysis of CFR adjusted for FFR.**

| **R^2^ = 0.159** | **Regression coefficient** | **Standard error** | **Standard regression coefficient** | **Test statistic** | **P value** | **95% confidence interval** | **VIF** |
| --- | --- | --- | --- | --- | --- | --- | --- |
| **Age** | **−0.036** | **0.020** | **−0.192** | **−1.803** | **0.075** | **−0.076 to 0.004** | **1.218** |
| **Sex** | **0.563** | **0.602** | **0.118** | **0.935** | **0.352** | **−0.633 to 1.759** | **1.704** |
| **BMI** | **0.108** | **0.057** | **0.214** | **1.872** | **0.064** | **−0.007 to 0.222** | **1.395** |
| **Mean blood pressure** | **−0.005** | **0.017** | **−0.033** | **−0.307** | **0.759** | **−0.038 to 0.028** | **1.267** |
| **Cr** | **0.831** | **1.313** | **0.079** | **0.633** | **0.528** | **−1.777 to 3.440** | **1.675** |
| **CRP** | **−1.115** | **0.832** | **−0.145** | **−1.340** | **0.184** | **−2.768 to 0.538** | **1.246** |
| **LDL-C** | **−0.015** | **0.008** | **−0.216** | **−1.795** | **0.076** | **−0.031 to 0.002** | **1.545** |
| **HbA1c** | **−0.973** | **0.375** | **−0.273** | **−2.594** | **0.011** | **−1.718 to −0.228** | **1.185** |
| **FFR** | **6.753** | **6.348** | **0.112** | **1.064** | **0.290** | **−5.859 to 19.365** | **1.195** |
| **Objective variable: CFR**  **Explanatory variables: Age, Sex, BMI, Mean blood pressure, CRP, LDL-C, FFR.** | | | | | | | |

BMI, body mass index; CFR, coronary flow reserve; Cr, creatinine; CRP, C-reactive protein; FFR, fractional flow reserve; HbA1c, hemoglobin A1c; LDL-C, low-density lipoprotein cholesterol; VIF, variance inflation factor.
