# Supplementary material for: Association between hemoglobin A1c levels and coronary flow reserve in angina with nonobstructive coronary arteries: a retrospective observational study
Source: BMC Cardiovasc Disord. 2026 Aug 10;26:1038. doi: 10.1186/s12872-026-06449-6 (PMC13741783; doi:10.1186/s12872-026-06449-6)
Supplement: Supplementary file 1 [file 12872_2026_6449_MOESM4_ESM.zip › Revised Supplementary Table S6.docx]

**Supplementary Table S6. Multiple regression analysis of CFR including HbA1c × sex interaction.**

| **R^2^ = 0.132** | **Regression coefficient** | **Standard error** | **Standard regression coefficient** | **Test statistic** | **P value** | **95% confidence interval** | **VIF** |
| --- | --- | --- | --- | --- | --- | --- | --- |
| **Age** | **−0.029** | **0.020** | **−0.160** | **−1.440** | **0.153** | **−0.069 to 0.011** | **1.340** |
| **Sex** | **0.582** | **0.583** | **0.124** | **0.998** | **0.321** | **−0.576 to 1.741** | **1.665** |
| **BMI** | **0.091** | **0.056** | **0.180** | **1.617** | **0.109** | **−0.021 to 0.202** | **1.345** |
| **Mean blood pressure** | **0.005** | **0.016** | **0.034** | **0.318** | **0.751** | **−0.026 to 0.037** | **1.211** |
| **Cr** | **0.962** | **1.294** | **0.092** | **0.743** | **0.459** | **−1.607 to 3.530** | **1.665** |
| **CRP** | **−0.875** | **0.813** | **−0.114** | **−1.076** | **0.285** | **−2.490 to 0.740** | **1.216** |
| **LDL-C** | **−0.013** | **0.007** | **−0.202** | **−1.808** | **0.074** | **−0.028 to 0.001** | **1.351** |
| **HbA1c** | **−0.890** | **0.426** | **−0.251** | **−2.090** | **0.039** | **−1.735 to −0.044** | **1.561** |
| **HbA1c× Sex** | **0.012** | **0.820** | **0.002** | **0.015** | **0.988** | **−1.615 to 1.640** | **1.584** |
| **Objective variable: CFR**  **Explanatory variables: Age, Sex, BMI, Mean blood pressure, CRP, LDL-C, HbA1c and HbA1c×Sex.** | | | | | | | |

BMI, body mass index; CFR, coronary flow reserve; Cr, creatinine; CRP, C-reactive protein; HbA1c, hemoglobin A1c; LDL-C, low-density lipoprotein cholesterol; VIF, variance inflation factor.
