# Supplementary material for: Association between hemoglobin A1c levels and coronary flow reserve in angina with nonobstructive coronary arteries: a retrospective observational study
Source: BMC Cardiovasc Disord. 2026 Aug 10;26:1038. doi: 10.1186/s12872-026-06449-6 (PMC13741783; doi:10.1186/s12872-026-06449-6)
Supplement: Supplementary file 1 [file 12872_2026_6449_MOESM4_ESM.zip › Revised Supplementary Table S1.docx]

**Supplementary Table S1. Endotype of ANOCA.**

| **Variable** | **n (%)** |
| --- | --- |
| **Acetylcholine provocation test performed** | **73 (70.2)** |
| **CSA** | **58 (55.8)** |
| **MVS** | **1 (1.0)** |
| **CMD** | **4 (3.8)** |
| **Combined CSA + CMD** | **2 (1.9)** |
| **No definitive diagnosis** | **39 (37.5)** |
| **Reduced CFR (<2.0)** | **12 (11.5)** |
| **Reduced CFR (<2.5)** | **23 (22.1)** |
| **Elevated IMR (≥25)** | **15 (14.4)** |

ANOCA: angina with nonobstructive coronary arteries, CFR: coronary flow reserve, CMD: coronary microvascular dysfunction, CSA: coronary spastic angina, IMR: index of microcirculatory resistance, MVS: microvascular spasm.
