# Supplementary material for: Association between hemoglobin A1c levels and coronary flow reserve in angina with nonobstructive coronary arteries: a retrospective observational study
Source: BMC Cardiovasc Disord. 2026 Aug 10;26:1038. doi: 10.1186/s12872-026-06449-6 (PMC13741783; doi:10.1186/s12872-026-06449-6)
Supplement: Supplementary file 1 [file 12872_2026_6449_MOESM4_ESM.zip › Revised Supplementary Table S5.docx]

**Supplementary Table S5. Multivariable logistic regression analysis for reduced coronary flow reserve (CFR <2.5).**

| **Variable** | **Odds ratio** | **95% CI** | **P value** |
| --- | --- | --- | --- |
| **Age** | **1.06** | **1.01 to 1.11** | **0.027** |
| **Sex** | **1.52** | **0.42 to 5.58** | **0.527** |
| **BMI** | **1.01** | **0.89 to 1.14** | **0.890** |
| **Mean blood pressure** | **0.98** | **0.95 to 1.02** | **0.339** |
| **Cr** | **0.21** | **0.01 to 4.79** | **0.329** |
| **CRP** | **0.84** | **0.15 to 4.78** | **0.847** |
| **LDL-C** | **1.01** | **1.00 to 1.03** | **0.153** |
| **HbA1c** | **1.85** | **0.82 to 4.16** | **0.136** |
| **Multivariable logistic regression was performed using the same covariates as in the primary multivariable linear regression model. Reduced CFR was defined as CFR <2.5.** | | | |

BMI, body mass index; CI, confidence interval; CFR, coronary flow reserve; Cr, creatinine; CRP, C-reactive protein; HbA1c, hemoglobin A1c; IMR, index of microcirculatory resistance; LDL-C, low-density lipoprotein cholesterol; OR, odds ratio.
