# Supplementary material for: Association between hemoglobin A1c levels and coronary flow reserve in angina with nonobstructive coronary arteries: a retrospective observational study
Source: BMC Cardiovasc Disord. 2026 Aug 10;26:1038. doi: 10.1186/s12872-026-06449-6 (PMC13741783; doi:10.1186/s12872-026-06449-6)
Supplement: Supplementary file 1 [file 12872_2026_6449_MOESM4_ESM.zip › Revised Supplementary Table S7.docx]

**Supplementary Table S7. Multiple regression analysis in men.**

| **R^2^ = 0.132** | **Regression coefficient** | **Standard error** | **Standard regression coefficient** | **Test statistic** | **P value** | **95% confidence interval** | **VIF** |
| --- | --- | --- | --- | --- | --- | --- | --- |
| **Age** | **−0.009** | **0.025** | **−0.055** | **−0.374** | **0.107** | **−0.059 to 0.041** | **1.329** |
| **BMI** | **−0.103** | **0.069** | **−0.216** | **−1.485** | **0.710** | **−0.242 to 0.037** | **1.289** |
| **Mean blood pressure** | **0.059** | **0.020** | **0.421** | **2.997** | **0.004** | **0.019 to 0.098** | **1.197** |
| **Cr** | **−1.913** | **1.416** | **−0.201** | **−1.351** | **0.183** | **−4.762 to 0.936** | **1.347** |
| **HbA1c** | **−0.545** | **0.372** | **−0.199** | **−1.467** | **0.149** | **−1.294 to 0.203** | **1.117** |
| **Objective variable: CFR**  **Explanatory variables: Age, Sex, BMI, Mean blood pressure, CRP, LDL-C, and HbA1c.** | | | | | | | |

BMI, body mass index; CFR, coronary flow reserve; Cr, creatinine; HbA1c, hemoglobin A1c; VIF, variance inflation factor.
