# Supplementary material for: Association between hemoglobin A1c levels and coronary flow reserve in angina with nonobstructive coronary arteries: a retrospective observational study
Source: BMC Cardiovasc Disord. 2026 Aug 10;26:1038. doi: 10.1186/s12872-026-06449-6 (PMC13741783; doi:10.1186/s12872-026-06449-6)
Supplement: Supplementary file 1 [file 12872_2026_6449_MOESM4_ESM.zip › Revised Supplementary Table S8.docx]

**Supplementary Table S8. Multiple regression analysis in women.**

| **R^2^ = 0.132** | **Regression coefficient** | **Standard error** | **Standard regression coefficient** | **Test statistic** | **P value** | **95% confidence interval** | **VIF** |
| --- | --- | --- | --- | --- | --- | --- | --- |
| **Age** | **−0.035** | **0.031** | **−0.177** | **−1.140** | **0.260** | **−0.097 to 0.027** | **1.359** |
| **BMI** | **0.076** | **0.084** | **0.145** | **0.912** | **0.366** | **−0.092 to 0.245** | **1.427** |
| **Mean blood pressure** | **−0.015** | **0.023** | **−0.091** | **−0.643** | **0.524** | **−0.061 to 0.031** | **1.127** |
| **Cr** | **3.798** | **2.772** | **0.208** | **1.370** | **0.177** | **−1.783 to 9.379** | **1.300** |
| **HbA1c** | **−0.628** | **0.809** | **−0.122** | **−0.776** | **0.442** | **−2.256 to 1.000** | **1.393** |
| **Objective variable: CFR**  **Explanatory variables: Age, Sex, BMI, Mean blood pressure, CRP, LDL-C, and HbA1c.** | | | | | | | |

BMI, body mass index; CFR, coronary flow reserve; Cr, creatinine; HbA1c, hemoglobin A1c; VIF, variance inflation factor.
