# Supplementary material for: Association between hemoglobin A1c levels and coronary flow reserve in angina with nonobstructive coronary arteries: a retrospective observational study
Source: BMC Cardiovasc Disord. 2026 Aug 10;26:1038. doi: 10.1186/s12872-026-06449-6 (PMC13741783; doi:10.1186/s12872-026-06449-6)
Supplement: Supplementary file 1 [file 12872_2026_6449_MOESM4_ESM.zip › Revised Supplementary Table S9.docx]

**Supplementary Table S9. Assessment of potential confounding by medication.**

**Medication use across HbA1c tertiles**

| **Variable** | **Tertile 1 (low)** | **Tertile 2 (middle)** | **Tertile 3 (high)** | **P value** |
| --- | --- | --- | --- | --- |
| **Statins** | **7 (21.2%)** | **16 (41.0%)** | **22 (68.8%)** | **<0.001** |
| **Ca-blockers** | **10 (30.3%)** | **12 (30.8%)** | **13 (40.6%)** | **0.604** |
| **β-blockers** | **7 (21.2%)** | **6 (15.4%)** | **10 (31.3%)** | **0.246** |
| **RAS inhibitors** | **14 (42.4%)** | **12 (30.8%)** | **16 (50.0%)** | **0.249** |

**Association with CFR**

| **Variable** | **β** | **P value** |
| --- | --- | --- |
| **Statins** | **−0.086** | **0.424** |
| **Ca-blockers** | **0.113** | **0.273** |
| **β-blockers** | **−0.052** | **0.642** |
| **RAS inhibitors** | **0.101** | **0.344** |

**Partial correlation analysis with possible affecting factors as control factors**

| **Control Variable** | **Independent Variables** | **Partial Correlation Coefficient** | **P Valve** |
| --- | --- | --- | --- |
| **Patients on Statins, Patients on β-blockers, Patients on Ca-blockers, Patients on RAS inhibitors** | **HbA1c ⇄ CFR** | **−0.220** | **0.028** |

CFR; coronary flow reserve, HbA1c; hemoglobin A1c, RAS inhibitors; renin-angiotensin system inhibitors.
