# Supplementary material for: Association between hemoglobin A1c levels and coronary flow reserve in angina with nonobstructive coronary arteries: a retrospective observational study
Source: BMC Cardiovasc Disord. 2026 Aug 10;26:1038. doi: 10.1186/s12872-026-06449-6 (PMC13741783; doi:10.1186/s12872-026-06449-6)
Supplement: Supplementary file 1 [file 12872_2026_6449_MOESM4_ESM.zip › Revised Supplementary Table S2.docx]

**Supplementary Table S2. Multiple regression analysis excluding patients treated with insulin.**

| **R^2^ = 0.129** | **Regression coefficient** | **Standard error** | **Standard regression coefficient** | **Test statistic** | **P value** | **95% confidence interval** | **VIF** |
| --- | --- | --- | --- | --- | --- | --- | --- |
| **Age** | **−0.032** | **0.020** | **−0.172** | **−1.585** | **0.116** | **−0.071 to 0.008** | **1.238** |
| **Sex** | **0.589** | **0.587** | **0.124** | **1.003** | **0.319** | **−0.578 to 1.756** | **1.615** |
| **BMI** | **0.089** | **0.056** | **0.178** | **1.590** | **0.115** | **−0.022 to 0.200** | **1.322** |
| **Mean blood pressure** | **0.007** | **0.016** | **0.043** | **0.407** | **0.685** | **−0.025 to 0.038** | **1.190** |
| **Cr** | **0.907** | **1.312** | **0.086** | **0.692** | **0.491** | **−1.698 to 3.513** | **1.640** |
| **CRP** | **−0.834** | **0.817** | **−0.109** | **−1.021** | **0.310** | **−2.457 to 0.789** | **1.205** |
| **LDL-C** | **−0.014** | **0.008** | **−0.211** | **−1.882** | **0.063** | **−0.029 to 0.001** | **1.325** |
| **HbA1c** | **−0.844** | **0.397** | **−0.223** | **−2.126** | **0.036** | **−1.632 to −0.056** | **1.164** |
| **Objective variable: CFR**  **Explanatory variables: Age, Sex, BMI, Mean blood pressure, Cr, CRP, LDL-C, and HbA1c.** | | | | | | | |

| **R^2^ = 0.172** | **Regression coefficient** | **Standard error** | **Standard regression coefficient** | **Test statistic** | **P value** | **95% confidence interval** | **VIF** |
| --- | --- | --- | --- | --- | --- | --- | --- |
| **Age** | **0.133** | **0.072** | **0.196** | **1.852** | **0.067** | **−0.010 to 0.275** | **1.238** |
| **Sex** | **1.615** | **2.111** | **0.092** | **0.765** | **0.446** | **−2.577 to 5.807** | **1.615** |
| **BMI** | **0.214** | **0.201** | **0.116** | **1.061** | **0.292** | **−0.186 to 0.614** | **1.322** |
| **Mean blood pressure** | **0.075** | **0.057** | **0.134** | **1.299** | **0.197** | **−0.040 to 0.189** | **1.190** |
| **Cr** | **6.658** | **4.714** | **0.172** | **1.412** | **0.161** | **−2.705 to 16.021** | **1.640** |
| **CRP** | **−5.234** | **2.936** | **−0.186** | **−1.782** | **0.078** | **−11.065 to 0.598** | **1.205** |
| **LDL-C** | **0.007** | **0.027** | **0.027** | **0.249** | **0.804** | **−0.047 to 0.061** | **1.325** |
| **HbA1c** | **2.746** | **1.426** | **0.197** | **1.926** | **0.057** | **−0.086 to 5.579** | **1.164** |
| **Objective variable: IMR**  **Explanatory variables: Age, Sex, BMI, Mean blood pressure, Cr, CRP, LDL-C, and HbA1c** | | | | | | | |

BMI, body mass index; CFR, coronary flow reserve; Cr, creatinine; CRP, C-reactive protein; HbA1c, hemoglobin A1c; IMR, index of microcirculatory resistance; LDL-C, low-density lipoprotein cholesterol; VIF, variance inflation factor.
