# Supplementary material for: Association between hemoglobin A1c levels and coronary flow reserve in angina with nonobstructive coronary arteries: a retrospective observational study
Source: BMC Cardiovasc Disord. 2026 Aug 10;26:1038. doi: 10.1186/s12872-026-06449-6 (PMC13741783; doi:10.1186/s12872-026-06449-6)
Supplement: Supplementary file 1 [file 12872_2026_6449_MOESM4_ESM.zip › Revised Supplementary Table S3.docx]

**Supplementary Table S3. Multiple regression analysis excluding patients with diabetes mellitus.**

| **R^2^ = 0.103** | **Regression coefficient** | **Standard error** | **Standard regression coefficient** | **Test statistic** | **P value** | **95% confidence interval** | **VIF** |
| --- | --- | --- | --- | --- | --- | --- | --- |
| **Age** | **−0.025** | **0.023** | **−0.137** | **−1.097** | **0.276** | **−0.071 to 0.020** | **1.286** |
| **Sex** | **0.144** | **0.700** | **0.029** | **0.205** | **0.838** | **−1.251 to 1.538** | **1.697** |
| **BMI** | **0.082** | **0.069** | **0.153** | **1.182** | **0.241** | **−0.056 to 0.220** | **1.383** |
| **Mean blood pressure** | **0.018** | **0.018** | **0.115** | **0.948** | **0.346** | **−0.019 to 0.054** | **1.216** |
| **Cr** | **−0.159** | **1.517** | **−0.015** | **−0.105** | **0.917** | **−3.181 to 2.863** | **1.741** |
| **CRP** | **−0.475** | **0.921** | **−0.064** | **−0.515** | **0.608** | **−2.309 to 1.360** | **1.272** |
| **LDL-C** | **−0.012** | **0.009** | **−0.172** | **−1.360** | **0.178** | **−0.029 to 0.005** | **1.314** |
| **HbA1c** | **−1.287** | **0.957** | **−0.165** | **−1.345** | **0.183** | **−3.193 to 0.620** | **1.239** |
| **Objective variable: CFR**  **Explanatory variables: Age, Sex, BMI, Mean blood pressure, Cr, CRP, LDL-C, and HbA1c.** | | | | | | | |

| **R^2^ = 0.142** | **Regression coefficient** | **Standard error** | **Standard regression coefficient** | **Test statistic** | **P value** | **95% confidence interval** | **VIF** |
| --- | --- | --- | --- | --- | --- | --- | --- |
| **Age** | **0.090** | **0.073** | **0.152** | **1.242** | **0.218** | **−0.055 to 0.235** | **1.286** |
| **Sex** | **−0.008** | **2.231** | **0.000** | **−0.003** | **0.997** | **−4.452 to 4.437** | **1.697** |
| **BMI** | **0.225** | **0.221** | **0.129** | **1.017** | **0.313** | **−0.215 to 0.665** | **1.383** |
| **Mean blood pressure** | **0.072** | **0.059** | **0.145** | **1.221** | **0.226** | **−0.045 to 0.189** | **1.216** |
| **Cr** | **3.999** | **4.833** | **0.118** | **0.827** | **0.411** | **−5.631 to 13.628** | **1.741** |
| **CRP** | **−4.132** | **2.934** | **−0.171** | **−1.409** | **0.163** | **−9.978 to 1.713** | **1.272** |
| **LDL-C** | **0.019** | **0.028** | **0.085** | **0.693** | **0.491** | **−0.036 to 0.074** | **1.314** |
| **HbA1c** | **3.325** | **3.049** | **0.131** | **1.090** | **0.279** | **−2.751 to 9.401** | **1.239** |
| **Objective variable: IMR**  **Explanatory variables: Age, Sex, BMI, Mean blood pressure, Cr, CRP, LDL-C, and HbA1c** | | | | | | | |

BMI, body mass index; CFR, coronary flow reserve; Cr, creatinine; CRP, C-reactive protein; HbA1c, hemoglobin A1c; IMR, index of microcirculatory resistance; LDL-C, low-density lipoprotein cholesterol; VIF, variance inflation factor.
